# Intramedullary Nails Result in More Reoperations Than Sliding Hip Screws in Two-part Intertrochanteric Fractures

**DOI:** 10.1007/s11999-012-2728-2

**Published:** 2012-12-07

**Authors:** Kjell Matre, Leif Ivar Havelin, Jan-Erik Gjertsen, Birgitte Espehaug, Jonas Meling Fevang

**Affiliations:** 1 Department of Orthopaedic Surgery, Haukeland University Hospital, Jonas Lies Vei 65, 5021 Bergen, Norway; 2 Department of Surgical Sciences, University of Bergen, Bergen, Norway; 3 Department of Health and Social Sciences, Bergen University College, Bergen, Norway

## Abstract

**Background:**

Sliding hip screws (SHSs) and intramedullary (IM) nails are well-documented implants for simple two-part intertrochanteric fractures; however, there is no consensus regarding which type of implant is better.

**Questions/purposes:**

We asked whether patients with simple two-part intertrochanteric fractures treated with IM nailing had (1) a lower reoperation rate and (2) less pain and better quality of life than patients treated with SHSs.

**Methods:**

We used data from the Norwegian Hip Fracture Register on 7643 operations for simple two-part intertrochanteric fractures (AO/OTA Type A1) treated with an SHS (n = 6355) or an IM nail (n = 1288) between 2005 and 2010. Kaplan-Meier analysis was used to assess reoperation percentages and a Cox regression model was used to assess the risk of reoperation. Questionnaires regarding pain and quality of life were answered by the patients at 4, 12, and 36 months postoperatively.

**Results:**

We found an increased risk of reoperation after IM nailing within 1 postoperative year: 2.4% and 4.2% for SHS and IM nails, respectively. The difference persisted with time: 4.5% and 7.1% at 3 years. We also found minor differences for pain and quality of life which we judged clinically unimportant.

**Conclusions:**

Based on our findings and a critical review of the literature, we suggest an SHS is likely the preferred implant for simple two-part intertrochanteric fractures.

**Level of Evidence:**

Level III, therapeutic study. See the Instructions for Authors for a complete description of levels of evidence.

## Introduction

Implant selection for intertrochanteric fractures remains controversial, and whether intertrochanteric fractures are best treated with a sliding hip screw (SHS) or an intramedullary (IM) nail has not been conclusively answered in the literature [17, 24]. Most randomized clinical trials (RCTs) [5, 23, 27, 29–31] found no major difference in long-term functional outcome between the two groups of implants. However, a meta-analysis [16] concluded higher fracture fixation failure and reoperation rates occurred after IM nailing. Jones et al. [16] concluded an IM nail should not be recommended for stable intertrochanteric fractures. Even for unstable fractures, they found no advantage in using an IM nail. Their findings, however, might have been skewed by the inclusion of studies on the earliest commercially available trochanteric nails and a learning curve among surgeons beginning to use trochanteric nailing. Some of the earlier nails were associated with higher failure rates, postoperative femoral fractures in particular, and are no longer in use [4, 8, 10, 25]. Bhandari et al. assessed the effects of time and different generations of implants (Gamma™ nails, Stryker, Kalamazoo, MI, USA) on femoral shaft fractures after nailing [6]. They found the differences in femoral fracture risk between the SHS and the Gamma™ nail lessened and eventually disappeared and therefore recommended the findings from earlier RCTs and meta-analyses should be interpreted with caution.

Thus, despite numerous publications on this topic, firm conclusions regarding the best implant for intertrochanteric fractures cannot be drawn and recommendations have diverged. In addition, a consistent fracture classification has not always been used, making the interpretation of data more difficult. Nevertheless, there has been a trend toward more IM nailing in intertrochanteric fractures, even though evidence supporting its increased use is missing [2, 26]. We have seen a similar but less pronounced trend in our country, but we still treat nearly 80% of all intertrochanteric fractures with an SHS [21].

To clarify the distinctions between these two implants, we studied a large group of patients with simple two-part fractures and specifically asked whether patients with simple two-part intertrochanteric fractures treated with IM nailing had (1) lower risks of reoperation and (2) less pain and better quality of life than patients treated with SHSs.

## Patients and Methods

**Fig. 1:**
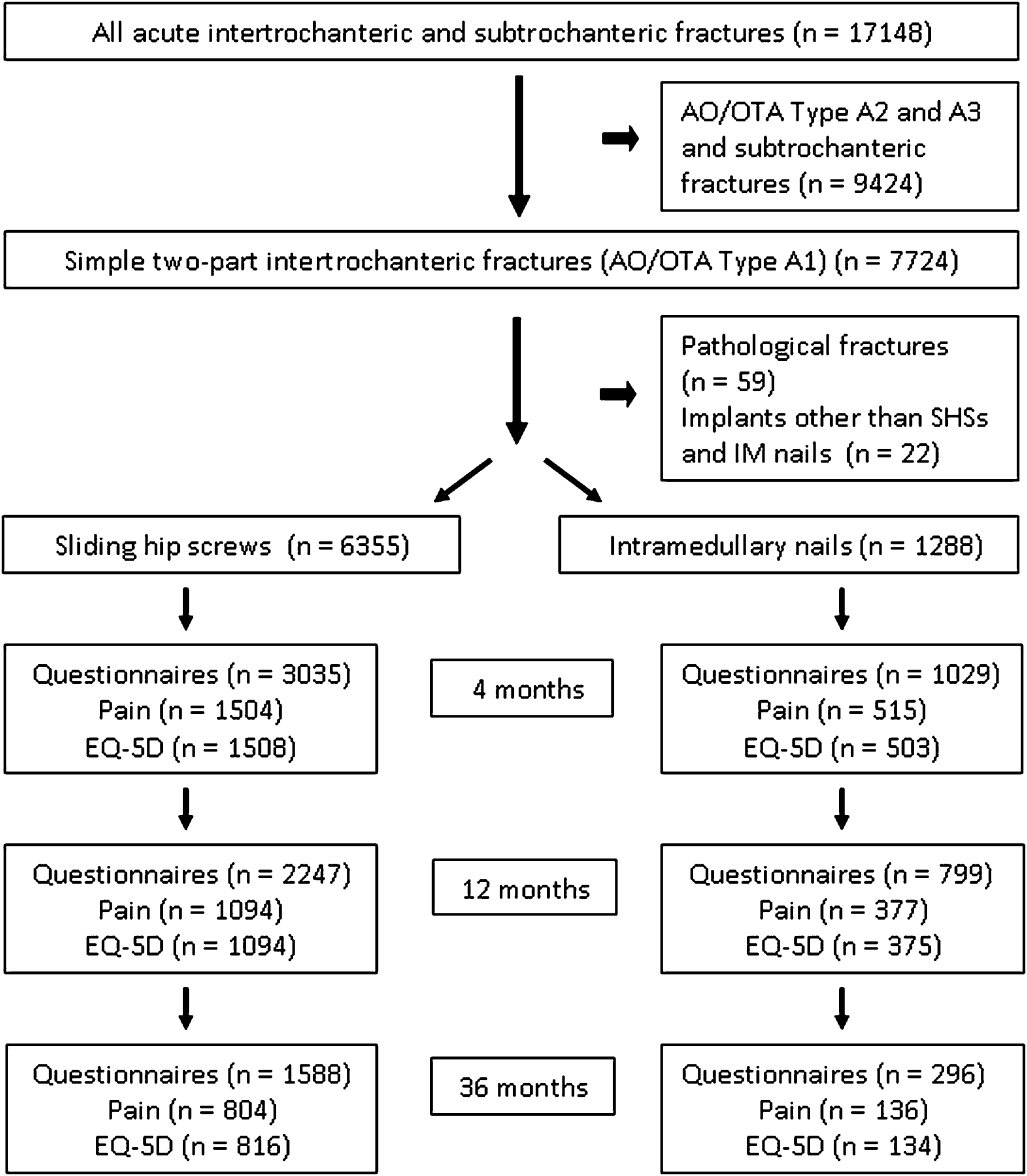
A flowchart of the patients and followup assessments is shown.

Since January 1, 2005, hip fracture operations in our country have been recorded prospectively in the Norwegian Hip Fracture Register (NHFR) [12]. Seventeen thousand one hundred forty-eight primary operations for intertrochanteric and subtrochanteric fractures were recorded until December 31, 2010. For the current study, we selected patients with two-part intertrochanteric fractures (AO/OTA Type A1 [19]) treated with an SHS or an IM nail (n = 7724). Operations performed with other implants (n = 22) and operations for pathologic fractures (n = 59) were excluded, leaving 7643 operations (6355 operations with SHSs and 1288 with IM nails) for final analyses (Fig. 1). The surgeons classified the fractures according to the AO/OTA classification and also reported the patients’ baseline characteristics (age, sex, cognitive function, American Society of Anesthesiologists [ASA] classification of morbidities) and details from the primary operations (surgical time, type of anesthesia, antibiotic and thrombotic prophylaxis). Overall, 71% of the patients were female, and the mean age for both groups was 82 years. We found no differences in the mean ASA scores, cognitive functions, or preoperative quality of life (EQ-5D™ index score; EuroQol Group, Rotterdam, The Netherlands) between the two treatment groups (Table 1).

**Table 1:** Baseline characteristics of the two groups

Characteristic	Sliding hip screw	Intramedullary nail	p value
Total number of hips (n = 7643)	6355 (83%)	1288 (17%)	
Age (years) (n = 7643)*	82 (10)	82 (10)	0.22^†^
Sex (number of hips) (n = 7643)			0.24^‡^
Female	4515 (71%)	936 (73%)	
ASA type (number of hips) (n = 7520)	6252	1268	0.007^‡^
1	463 (7%)	66 (5%)	
2	2224 (36%)	506 (40%)	
3	3216 (51%)	629 (50%)	
4	337 (5%)	66 (5%)	
5	12 (0.2%)	1 (0.1%)	
ASA score*	2.55 (0.7)	2.55 (0.7)	0.88^†^
Cognitive impairment (number of hips) (n = 7453)	6198	1255	0.10^‡^
Yes	1522 (25%)	288 (23%)	
No	4009 (65%)	808 (64%)	
Uncertain	667 (11%)	159 (13%)	
Preoperative EQ-5D™ index score* (n = 2038)	0.69 (0.28)	0.69 (0.29)	0.71^†^
Surgical time (minutes)* (n = 7643)	52 (25)	51 (23)	0.029^†^
Anesthesia (n = 7643)			0.67^‡^
Spinal	90%	90%	
General	6%	6%	
Other or missing	4%	4%	
Antibiotic prophylaxis (n = 7643)			< 0.001^‡^
Yes	95%	86%	
No	5%	13%	
Missing value	0.6%	0.8%	
Thrombosis prophylaxis	99%	99%	0.63^‡^

* Values are expressed as mean, with SD in parentheses; ^†^Student’s t-test; ^‡^Pearson chi-square test; ASA = American Society of Anesthesiologists.

Power calculations, including the number of patients in the SHS and IM nail groups (6355 and 1288, respectively), were performed. We considered a difference in reoperation percentages of 1% to 2% to be clinically relevant, and detecting a significant difference in reoperations of 2% could be obtained with a power of 85% by using our numbers of patients. Accordingly, our study had sufficient power to detect a clinically important difference of this size.

The SHS has remained the most commonly used implant in Norway for treatment of all intertrochanteric and subtrochanteric fractures [21]. In our study, compression hip screws (AMBI^®^/CLASSIC Hip Screw System; Smith & Nephew, London, UK) and dynamic hip screws (Dynamic Hip System screw/blade; Synthes GmbH, Basel, Switzerland) were the two most frequently used SHSs. A trochanteric stabilizing plate was added in 8% of these operations, possibly to prevent fracture of a small and osteoporotic lateral spike of the trochanter at mobilization. The second and third generations of the Gamma3™ Locking Nail (Stryker Corp) and the Trigen™ Intertan™ Trochanteric Antegrade Nail (Smith & Nephew) were the most commonly used IM nails. Long nails were used in 4% of the nailing procedures (Table 2).

**Table 2:** Implants used

Implant	Number of hips
Sliding hip screws	
Compression hip screw (AMBI^®^/CLASSIC Hip Screw System)*	3887 (61%)
Dynamic hip screw (Dynamic Hip System)^†^	1929 (30%)
Locking compression plate (Dynamic Hip System)^†^	492 (8%)
Omega Plus™^‡^	43 (0.7%)
Other/missing data	4 (0%)
Total	6355 (100%)
Intramedullary nails	
Gamma3™ Locking Nail^‡^	699 (54%)
Trigen™ Intertan™*	355 (28%)
Trochanteric-Gamma™^‡^	154 (12%)
Proximal femoral nail-antirotation^†^	51 (4%)
Proximal femoral nail^†^	11 (0.9%)
Intramedullary hip screw*	10 (0.8%)
Other nails/missing data	8 (0.6%
Total	1288 (100%)

* Smith & Nephew, London, UK; ^†^Synthes, Basel, Switzerland; ^‡^Stryker Corp, Kalamazoo, MI, USA.

Operating surgeons from 55 hospitals nationwide reported primary operations and reoperations, with causes and type of reoperation, to the NHFR. Failure of the fixation, nonunions or malunions, femoral head necroses, local pain from protruding hardware, infections, hematomas, cutouts, periimplant fractures, and other occurrences were the options for reporting causes of reoperation. Removal of the implants, resection arthroplasties, unipolar or bipolar hemiarthroplasties, refixation, débridement for infections, and other occurrences were the options for reporting type of reoperations. More than one cause of reoperation and more than one type of reoperation were recorded for some patients. Patients whose reoperations were THAs (n = 81), however, were reported to the Norwegian Arthroplasty Register. The NHFR obtained these data and linked them to the primary operations, but we had no detailed information regarding the causes of reoperations for these patients.

Questionnaires regarding quality of life (EQ-5D™ health questionnaire) [28] and pain were sent to the patients at 4, 12, and 36 months postoperatively. A preoperative quality-of-life status was recorded in retrospect together with the 4-month questionnaire. At 4 months, 1029 patients with an IM nail received the questionnaires, and 515 and 503 answered the questionnaires regarding pain and EQ-5D™, respectively, giving a response rate of approximately 50% (Fig. 1). In the questionnaires, the patients were asked to report pain from the surgically treated hip, using a VAS (0 indicating no pain, 100 indicating unbearable pain). The EQ-5D™ questionnaire contains five factors (mobility, degree of self-care, ability to perform usual activities, pain/discomfort, and anxiety/depression) rated at three levels (no problems, some problems, severe problems). Derived from these questions, the EQ-5D™ index score gives a value, with a maximum score of 1.0 indicating a very good quality of life and a score of 0 being equivalent to death.

All patients were observed for any reason for reoperation until December 31, 2010 (mean followup, 1 year 10 months; range, 0–6 years). The questionnaires regarding pain and quality of life were sent to all living patients with IM nails or SHSs with a trochanteric stabilizing plate during followup from 2005 to 2010. Similarly, all patients with simple SHS operations in 2005, 2006, and 2010 received this questionnaire. Of the patients treated with a simple SHS in 2007 to 2009, however, owing to lack of resources, only a randomly selected subgroup of patients was asked to answer the questionnaires.

We estimated the cumulative 1- and 3-year reoperation risks for the two treatment groups using a Kaplan-Meier survival analysis. The log-rank test was used to detect differences. Patients without reoperations were censored at their dates of death or emigration or at the end of followup (December 31, 2010). The National Population Register provided death and emigration information. In addition, relative differences in reoperation rates (relative risk [RR]) between the implant types were estimated in a multiple Cox regression model with adjustments for possible confounding factors (age, sex, ASA class, cognitive impairment). Patients without complete information regarding their ASA classes and cognitive impairments (n = 290) were excluded from the regression analysis. The mortality during followup was determined with Kaplan-Meier analyses. Differences in mean pain and quality of life (EQ-5D™ index score) scores were analyzed using Student’s t-test, while categorical outcome variables (EQ-5D™ mobility and usual activity) were analyzed using the Pearson chi-square test. We used PASW^®^ Statistics Software (Version 18.0; SPSS Inc, Chicago, IL, USA) for all statistical analyses.

## Results

**Fig. 2:**
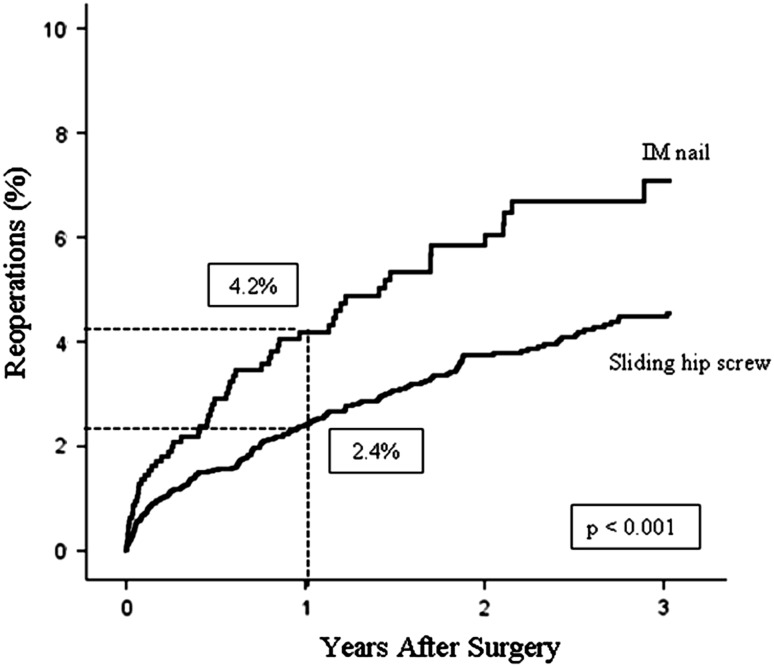
Kaplan-Meier analysis found cumulative reoperation rates of 4.2% and 2.4% at 1 year and 7.1% and 4.5% at 3 years for IM nails and SHSs, respectively.

We found a higher (p = 0.001) 1-year reoperation rate for patients treated with IM nails than for those treated with SHSs (4.2% and 2.4%, respectively). Two-hundred forty-nine reoperations were identified. At 3 years, the reoperation rates were 7.1% for IM nails and 4.5% for SHSs (p < 0.001) (Fig. 2). There was an overall 61% increased (p = 0.002) risk of reoperation after IM nailing, compared with that after using an SHS (RR, 1.61; 95% CI, 1.19–2.17). Comorbidity (ASA class) and sex did not influence the reoperation rates, whereas cognitively impaired patients had a lower (p < 0.001) reoperation risk than those who were cognitively lucid (RR, 0.44; 95% CI, 0.28–0.68). In addition, older (p = 0.049) age reduced the reoperation risk (Table 3). Failure of the fixation was the most common reason for reoperation in both groups (0.8%), and we found no differences between the two groups for most reasons for reoperations. However, the rates of periimplant fractures (p = 0.027) and reoperations attributable to implant-related pain (p = 0.043) were higher in the IM nail group. Accordingly, implant removal was more frequent (p = 0.028) in that group. Otherwise, the distribution of types of reoperations was similar for the two groups, but reoperations in the SHS group more frequently were recorded with a combination of reasons for reoperation (not just one reason) (Table 4). We found a higher (p = 0.016) reoperation rate for the 52 patients with a long nail in our study (six of 52 versus 54 of 1236).

**Table 3:** Cox regression analysis of factors with possible influences on the risk of reoperation

Factor	Relative risk	95% CI	p value
Type of implant			
Sliding hip screw	1		
Intramedullary nail	1.61	1.19–2.17	0.002
Sex			
Male	1		
Female	1.11	0.82–1.49	0.51
Age*	0.99	0.98–1.00	0.049
ASA type			
1	1		
2	1.07	0.69–1.67	0.76
3	0.93	0.59–1.45	0.74
4	1.12	0.52–2.42	0.77
Cognitive impairment			
No	1		
Uncertain	0.79	0.50–1.24	0.31
Yes	0.44	0.29–0.69	< 0.001

Patients were followed until reoperation, end of study inclusion, time of emigration, time of patient’s death; * risk reduction for each year of older age; ASA = American Society of Anesthesiologists.

**Table 4:** Reason for and type of reoperation versus type of implant in 249 hips with reoperations

Reoperations	Number of hips		p value*
	Sliding hip screw	Intramedullary nail	
Reoperated hips (overall 249/7643 [3.3%])	189/6355 (3.0%)	60/1288 (4.7%)	0.002
Reported reasons^†^			
Failure of osteosynthesis	54 (0.8%)	10 (0.8%)	0.79
Nonunion	18 (0.3%)	2 (0.2%)	0.41
Local pain from implant	17 (0.3%)	8 (0.6%)	0.043
Infection (deep and superficial)	14 (0.2%)	2 (0.2%)	0.64
Cutout	17 (0.3%)	7 (0.5%)	0.11
Fracture around implant	10 (0.2%)	6 (0.5%)	0.027
Other reasons	31 (0.5%)	12 (0.9%)	0.05
Unknown reasons (THAs^‡^)	63 (1.0%)	18 (1.4%)	0.19
Types of reoperations^§^			
Implant removal	25 (0.4%)	11 (0.9%)	0.028
New osteosynthesis	35 (0.6%)	10 (0.8%)	0.33
Bipolar hemiarthroplasty	50 (0.8%)	16 (1.2%)	0.11
THA	63 (1.0%)	18 (1.4%)	0.19
Debridement for infection	17 (0.3%)	3 (0.2%)	0.83
Others	8 (0.1%)	3 (0.2%)	0.36

* Pearson chi-square test; ^†^more than one reason per reoperation possible; 208 reasons for reoperations were reported in 249 hips; ^‡^for the 81 patients whose reoperation was a THA, no detailed descriptions of reasons for the reoperations were given; ^§^reporting more than one type of procedure was possible for each reoperation.

The average scores for pain were similar for the two implant groups at all times during the followup (Table 5). Four months postoperatively, the mean VAS pain scores were 28 and 29 for the IM nail and SHS, respectively (p = 0.332); they then decreased to 22 and 23, respectively, 3 years postoperatively (p = 0.845). We found no major differences between the two treatment groups in the quality-of-life assessments (Table 5). After analyzing the five factors of the EQ-5D™ questionnaire separately, however, we found, after 1 postoperative year, patients in the SHS group reported more problems regarding their mobility and performing usual activities.

**Table 5:** Pain and quality of life (with selected subcategories) in the two groups

Variable	Sliding hip screw	Intramedullary nail	Mean difference (95% CI)	p value
Mean VAS score for pain (points)				
4 months	29 (n = 1504)	29 (n = 515)	0.9 (−1.2 to 3.1)	0.40
1 year	26 (n = 1097)	24 (n = 378)	1.7 (−0.8 to 4.1)	0.19
3 years	23 (n = 804)	22 (n = 136)	0.4 (−3.3 to 4.0)	0.85
Mean EQ-5D™ index score*				
Preoperative	0.69 (n = 1519)	0.69 (n = 519)	0.005 (−0.023 to 0.034)	0.71
4 months	0.49 (n = 1508)	0.51 (n = 503)	−0.017 (−0.045 to 0.009)	0.20
1 year	0.55 (n = 1097)	0.58 (n = 376)	−0.030 (−0.061 to 0.001)	0.06
3 years	0.59 (n = 816)	0.59 (n = 134)	−0.008 (−0.061 to 0.044)	0.76
EQ-5D™: mobility at 12 months^†^				0.006
No problems	24%	32%		
Some problems	72%	65%		
Severe problems	4%	4%		
EQ-5D™: usual activities at 12 months^†^				0.014
No problems	26%	33%		
Some problems	47%	43%		
Severe problems	27%	24%		

* EQ-5D™ index score scale: 0 indicates a situation similar to death and 1 indicates the best possible quality of life; †no significant differences were found at 4 months or 3 years or for other EQ-5D™ dimensions at any time.

We also found the average surgical times for the two operative methods were almost identical: 52 minutes for the SHS group and 51 minutes for the IM nail group (p = 0.029). Mortality rates after 1 postoperative year were 25% for the SHS group and 23% for the IM nail group (p = 0.224).

## Discussion

There has been a trend toward more IM nailing in intertrochanteric fractures, but this trend has not been based on current evidence [2, 26]. Historically, higher failure rates have been observed after IM nailing compared with operations using SHSs [6, 16, 24]. To what extent modern nails reduce complication rates or improve function (if at all) remains to be shown. Currently, there is no consensus regarding which implant, an SHS or an IM nail, is the best for different intertrochanteric fractures. We therefore asked whether patients with simple two-part intertrochanteric fractures treated with IM nailing had (1) lower reoperation rates and (2) less pain and better quality of life than patients treated with SHSs.

There were some limitations to our study. First, as there had been no randomization of the treatment allocation, patient- and surgeon-related confounders may have been present. With comparable baseline characteristics for the groups, however, we believe the risk of any important bias is less likely. In addition, data representing a national average of hospitals and surgeons and the fact that the implant selection usually reflects the policy in each hospital rather than the choice of each surgeon should have reduced the chance of bias. Second, our responder rate was low, partly because of high mortality rates and the elderly study population, but the large number of included patients may have, to some extent, compensated for this. Underreporting of complications and reoperations might be anticipated. Even so, this probably should have affected both treatment groups equally, and most likely, the difference in the reoperation rates was real. Third, different IM nails and SHSs were used in our study, and we did not examine pain, function, or reoperation rates for each implant brand. Therefore, our results may not be generalized to any nail or SHS. Fourth, as the fracture classification is performed by the operating surgeons, and we have no radiographs available in our register, this is also a source of uncertainty. Finally, in a register study including thousands of patients, even minor and clinically irrelevant differences might become statistically significant. Accordingly, our data should be interpreted with caution. Nevertheless, where RCTs may fail to detect small differences owing to limited numbers of patients in rare events like reoperations in particular, we believe the large number of patients in a register study can add valuable information [14].

We found a higher rate of complications and reoperations after IM nailing than after SHS operations for simple two-part trochanteric fractures. Reoperation percentages of 2.4% and 4.2% for the SHS and IM nail groups at 1 year were comparable to rates in other reports [1, 3, 16] on intertrochanteric fractures. In line with our results, one meta-analysis of RCTs [16] concluded the failure rate were higher after IM nailing of stable intertrochanteric fractures than after using an SHS, and nailing of these fractures was not recommended. Our reoperation rates were slightly higher than those reported for stable fractures in that review but were lower than those reported in other studies [1, 3, 22] where stable and unstable fractures were not separated. Even though absolute numbers of reoperations vary among studies, the consistent overall difference in favor of the SHS seems to have remained. The severity of the complications and reasons for reoperation may vary among implant groups. In our study we found more patients had reoperations because of fracture around the implant and local pain from the implant in the IM nail group. Otherwise we found no differences in reasons for reoperation between the groups, indicating a similar rate of minor or major complications in both groups. Most types of reoperations were more frequent in the IM nail group, however, only “removal of implants” was significant. Postoperative femoral fracture rates were high when using the first few generations of IM nails [4, 8, 10, 25]. Therefore, reported failure rates after IM nailing, including nails no longer in use, may distort the results in updated reviews [15, 18, 24]. This problem was addressed in a meta-analysis by Bhandari et al. [6] who assessed the change of postoperative femoral fracture rates after Gamma™ nailing with time. They found less femoral fractures and no differences compared with the SHS in the most recent studies. However, no studies published after 2005 or studies on other types of IM nails were included in that review. In addition, others did not find a similar time-dependent change in the postoperative femoral fracture and failure rates for IM nailing [7, 24]. We suspect some underreporting of femoral fractures and subsequent reoperations in our study, as only six reoperations (0.5%) in the IM nail group were caused by fractures around the implants. These findings contrast with those in another study [11], where a 6% rate of postoperative femoral fractures was reported after IM nailing, clearly indicating this problem has not been solved. Our data included only recent generations of implants and indicated reoperation rates have continued to be higher after IM nailing of simple two-part intertrochanteric fractures. In our study, 96% of the nailing procedures were performed with short nails, and to what extent a shift toward more long nails even in stable intertrochanteric fractures would reduce the number of periimplant fractures remains unknown. However, despite a higher rate of reoperations for long nails, periimplant fractures were not the cause of reoperation in patients who were treated with long nails. We found the reoperation rate among cognitively impaired patients to be lower than that for cognitively lucid patients. This is consistent with another report [13] from our hip fracture register and might be caused by these patients’ poorer abilities to express complaints and/or differences in the indications for surgical interventions.

We also found no difference in pain or quality of life between the two implant groups during followup. The assessment of pain for patients with hip fractures has not been standardized, and several outcomes for pain have been reported [9, 24]. Therefore, comparing results is difficult. Nevertheless, regardless of the implant and outcome measure used and in accordance with our results, two meta-analyses [9, 24] reported no major differences in pain between implants and operative methods in trochanteric fractures. Our finding of no difference in the reported quality of life between the implants, using the EQ-5D™ index score, indicated the difference in reoperation rates was not enough to influence the patients’ perception of quality of life. After 1 postoperative year, however, more patients in the IM nail group rated their mobility and ability to perform usual activities with the best score. The differences were minor and temporary, but these EQ-5D™ dimensions describe important factors related to a patient’s ability to maintain his or her independence. Quality-of-life measures have been reported inconsistently in trials comparing the SHS and IM nail in intertrochanteric fractures [9]. We were not aware of any other study assessing quality of life using the EQ-5D™ questionnaire in cases of simple two-part intertrochanteric fractures. In a RCT comparing the Gamma™ nail with the Medoff sliding plate (Swemac, Linköping, Sweden) in unstable intertrochanteric and subtrochanteric fractures [20], the authors reported no difference in EQ-5D™ index scores between the groups. Overall, the most updated and thorough review of RCTs [24] comparing SHSs and IM nails in intertrochanteric fractures concluded there was no difference in terms of quality-of-life issues, such as pain, walking ability, or the number of patients regaining their prefracture levels of independence after intertrochanteric fractures.

We found a higher rate of reoperations after IM nailing than after use of the SHS in simple two-part intertrochanteric fractures, but we also found no clinically relevant differences in pain or overall quality of life during the followup assessments. Our study had several limitations, but the findings seemed to be in accordance with meta-analyses of RCTs. Despite modern trends suggesting otherwise, in our opinion, the SHS still seems to be the better treatment for simple two-part intertrochanteric fractures compared with short IM nails.
